# The biological impacts of CEBPD on urothelial carcinoma development and progression

**DOI:** 10.3389/fonc.2023.1123776

**Published:** 2023-01-27

**Authors:** Ti-Chun Chan, Yow-Ling Shiue, Chien-Feng Li

**Affiliations:** ^1^ Department of Medical Research, Chi Mei Medical Center, Tainan, Taiwan; ^2^ National Health Research Institutes, National Institute of Cancer Research, Tainan, Taiwan; ^3^ Institute of Precision Medicine, National Sun Yat-Sen University, Kaohsiung, Taiwan; ^4^ Department of Clinical Medicine, Chi Mei Medical Center, Tainan, Taiwan

**Keywords:** CEBPD, urothelial carcinoma, chromosomal instabilities, glycolysis, resistance

## Abstract

Urothelial carcinoma (UC), which includes urinary bladder urothelial carcinoma (UBUC) and upper tract urothelial carcinoma (UTUC), is one of the most common malignancies worldwide. Accordingly, a comprehensive understanding of the underlying mechanism governing UC development is compulsory. Aberrant CCAAT/enhancer-binding protein delta (CEBPD), a transcription factor, displays an oncogene or tumor suppressor depending on tumor type and microenvironments. However, CEBPD has been reported to possess a clear oncogenic function in UC through multiple regulation pathways. Genomic amplification of *CEBPD* triggered by MYC-driven genome instability is frequently examined in UC that drives CEBPD overexpression. Upregulated CEBPD transcriptionally suppresses *FBXW7* to stabilize MYC protein and further induces hexokinase II (HK2)-related aerobic glycolysis that fuels cell growth. Apart from the MYC-dependent pathway, CEBPD also downregulates the level of hsa-miR-429 to enhance HK2-associated glycolysis and induce angiogenesis driven by vascular endothelial growth factor A (VEGFA). Additionally, aggressive UC is attributed to the tumor metastasis regulated by CEBPD-induced matrix metalloproteinase-2 (MMP2) overexpression. Furthermore, elevated CEBPD induced by cisplatin (CDDP) is identified to have dual functions, namely, CDDP-induced chemotherapy resistance or drive CDDP-induced antitumorigenesis. Given that the role of CEBPD in UC is getting clear but pending a more systemic reappraisal, this review aimed to comprehensively discuss the underlying mechanism of CEBPD in UC tumorigenesis.

## Introduction

### The general concepts of urothelial carcinoma

Urothelial carcinoma (UC), also referred to as transitional cell carcinoma, is one of the most common malignancies worldwide. The majority of UC, approximately 90%–95%, resides in the bladder organ called urinary bladder urothelial carcinoma (UBUC). In 2020, UBUC was ranked as the 10th most frequently diagnosed cancer with 573,000 new cases and 213,000 new deaths around the world estimated by the World Health Organization (WHO) (1). Men significantly have four times higher risk compared with women to develop UBUC (2). Another relatively rare type of UC called upper tract urothelial carcinoma (UTUC) that accounts for 5%–10% of UC is located in the renal pelvis and ureter (3). The annual incidence of UTUC ranges from 1.2 to 4.7 cases per 100,000 inhabitants and also frequently occurs in men globally (4). In contrast, UTUC has an unusually high incidence that accounts for 40% of UC in Taiwan (5). The incidence rate of UTUC was 4.21 cases per 100,000 women, which was higher than those of men with 3.61 cases per 100,000 men based on the Health Promotion Administration of Taiwan in 2016 (6). According to the depth of invasion, UBUC can be divided into non-muscle-invasive bladder cancer [NMIBC; stages carcinoma *in situ* (CIS), Ta, T1) or muscle-invasive bladder cancer (MIBC; stages T2, T3, T4) (7). Approximately 70% of newly diagnosed UBUC are NMIBC with roughly 70% as Ta, 20% as T1, and 10% as Tis (8). In the grading system, Ta is divided into papillary urothelial neoplasms of low malignant potential (PUNLMP), low-grade noninvasive papillary urothelial carcinoma, and high-grade noninvasive papillary urothelial carcinoma. By definition, CIS and the greater part of T1 belong to high-grade (9). In general, NMIBC is considered to have low metastasis and a favorable prognosis with 90% 5-year overall survival rates. However, a high recurrence rate of up to 50% after transurethral resection of the bladder tumor (TURBT) and the propensity of CIS progression to an invasive stage largely challenge the treatment of NMIBC (10, 11). In contrast, MIBC is characterized as rapid metastatic progression and subsequently high mortality with 60% and 6% 5-year survival rate at stages T2 and T4, respectively (12, 13).

### Causal factors of UC initiation and progression

UBUC and UTUC share some of the same carcinogenic factors such as cigarette smoking and industrially hazardous chemicals (14). Smokers have 2.5–7 times more risk to develop UBUC, the same as UTUC, compared with nonsmokers (15). However, other particular carcinogens such as aristolochic acid (AA) are responsible for an unusually high prevalence of UTUC in Taiwan and some rural regions of southeastern Europe on account of Chinese herb remedy and soil contamination with *Aristolochia clematitis*, respectively (16, 17). Additionally, substantial pieces of evidence verified genetic predisposition to the development of UC. Genome-wide association study (GWAS) manifests that several suspicious SNPs of *SLC14A1*, a urea transporter UT-B mapped to chromosome 18q12.3, is strongly related to UBUC (18). Overall, in UTUC and UBUC, the landscape of aberrant genes is similar but a difference exists in the prevalent mutation. High-grade UTUC has a higher tendency for genomic alteration in fibroblast growth factor receptor 3 (FGFR3), Hras proto-oncogene, GTPase (*HRAS*), and cyclin-dependent kinase inhibitor 2B (*CDKN2B*), whereas high-grade UBUC is more frequently altered in tumor protein p53 (*TP53*), AT-rich interaction domain 1A (*ARID1A*), RB transcriptional corepressor 1 (*RB1*), and erb-b2 receptor tyrosine kinase 2 (*ERBB2*) (19, 20). Furthermore, two divergent carcinogenic pathways (i.e., the papillary and the non-papillary pathways) are used to explain the evolution of UBUC. The papillary pathway is driven by point mutations in *FGFR3*, *RAS*, and *PIK3CA* (phosphatidylinositol-4,5-bisphosphate 3-kinase catalytic subunit alpha) to develop hyperplasia, low-grade Ta, and high-grade Ta/T1 through constitutive activation of RTK/RAS/RAF/mitogen-activated protein kinase (MAPK) and phosphoinositide 3-kinase (PI3K)/Akt/mammalian target of rapamycin (mTOR) pathways related to cell proliferation (21, 22). Instead, the nonpapillary pathway is defined by the loss-of-function mutation in genes associated with DNA replication/repair machinery such as p53 and RB that gives rise to the progression of dysplasia, Tis to MIBC (23). Moreover, loss of heterozygosity (LOH) in chromosome 9 harboring various tumor suppressor genes such as *CDKN2A* (9p21), *TSC1* (9q34), and *PTCH1* (9q22) exists in high frequency in both hyperplasia and dysplasia, suggesting that copy number alteration of chromosome 9 could be the necessary early event toward the initiation of UBUC (24).

### Common chromosomal aberrations in UC

Frequent genetic copy number aberration is an important cancer hallmark strongly associated with carcinogenesis and aggressiveness (25). Aside from chromosome 9, recurrent chromosomal abnormalities in UC were also identified among distinct populations. Using tiling-resolution 32K BAC arrays to conduct genome-wide DNA copy number profiling pointed that copy number gains (CNGs) at chromosomes 1q, 3p, 3q, 5p, 6p, 8q, 18p, 20p, and 20q and copy number loss (CNL) at chromosomes 2q, 5q, 8p, 9p, 9q, 10q, 11p, 13q, 17p, and 22q are identified in the 146 UC specimens from Skåne University Hospital, Sweden (26). Using 40 UBUC samples from Taiwan that were submitted to array comparative genomic hybridization (aCGH) showed that CNG appears at chromosomes 1q, 3q, 5p, 7p, 10p, 11q, 17q, 18p, 19q, 20p, 20q, and 22q, while CNL exists at chromosomes 2q, 3p, 4q, 5q, 6q, 7q, 8p, 9q, 10q, 11p, 13q, 17p, and 18q. Among these genetic aberrations, the frequent chromosomal instability mainly resides in -5q, -9p, and +8q, corresponding to the study by Lindgren et al. (26). Additionally, CNG of chromosome 8q behaved as the most predominant amplification core and tended to result in poor clinical outcomes with developed disease-specific death [dead of disease (DOD)] and distal metastasis (DM) in UC patients than non-event cases (27). Mounting studies have proven that the amplification of *MYC* mapped to 8q24.21 is the major measure to prompt MYC overexpression and is regularly examined in aggressive cancers including UC with metastasis and unfavorable survival outcome (25, 28). Moreover, CEBPD overexpression driven by amplified *CEBPD* mapped to 8q11.21 in UC was pointed to have a remarkable predisposition toward the worst prognosis outcomes concerning disease-specific survival (DSS) and metastasis-free survival (MeFS) (27).

### MYC-induced genome instability potentiates *CEBPD* aberration in UC

CEBPD, a versatile transcription factor, belongs to the C/EBP family that comprises three parts: an N-terminal transactivating region, a basic DNA binding region, and a C-terminal leucine-zipper domain (29, 30). Typically, the CEBPD level is relatively low in many normal physiological statements while it is rapidly elevated by outside stimulations such as immune activation and inflammatory response (31). CEBPD presents as a regulator in terms of cell differentiation, proliferation, motility, growth arrest, and cell death (32). Over the past few years, accumulating research has identified the dual functions of CEBPD as a tumor suppressor in pancreatic ductal adenocarcinoma (PDAC), hepatocellular carcinoma, and breast cancer but an oncogene in glioblastoma and UC, relying on the tumor microenvironment and cell types (27, 33–36). Accordingly, this review intended to comprehensively summarize the regulatory mechanism on initial CEBPD overexpression followed by UC progression and cisplatin (CDDP)-resistant impact.

Previous studies elucidated that epigenetic events such as CpG island hypermethylation get involved in the silence of CEBPD and are associated with metastasis of breast cancer (37). Interestingly, the methylation level at the *CEBPD* promoter region is usually low and has no difference among the normal epithelium and low and high CEBPD-expressing UC tissues and UC cell lines (HT1197, TCCSUP, BFTC905, BTFC909, J82, RT4), indicating that the effect of CEBPD level is irrelevant to aberrant DNA methylation in UC. Furthermore, the high expression of CEBPD observed in the UC is attributed to the transcriptional activity of *CEBPD* amplification conferred by the low frequent promoter methylation (27). Intriguingly, UBUC specimens with a specifically gained region on chromosome 8q represented that *MYC* CNG is usually concurrent with *CEBPD* CNG and a positive correlation significantly exists between *MYC* and *CEBPD* gene dosage/transcripts. Noticeably, only MYC overexpression provoked *CEBPD* CNG, but CEBPD overexpression failed to cause *MYC* CNG in UC cells, indicating that a substantial MYC level occurred prior to *CEBPD* genome instability (38). Aberrant MYC is well known to set off genomic instability followed by cancer initiation (39). An abundant MYC level triggered by *MYC* amplification has been identified to amplify oncogene *ERBB2* (erb-b2 receptor tyrosine kinase 2) mapped to chromosome 17q12 and *DHFR* (dihydrofolate reductase) mapped to chromosome 5q14.1 to strengthen cell proliferation and high metastatic risk (40, 41). Using next-generation sequencing (NGS)-based loss of heterozygosity (LOH) assay indicated that the MYC overexpression significantly induces genomic LOH in distinct UC cell lines. The NGS-based Hi-C experiment, a method to investigate such long-range interactions between two different loci and important for transcriptional modulation of genes, pointed out that there is no obvious chromosomal contact between *MYC* and *CEBPD* loci with 80 Mb apart from each other. The abovementioned results fortified that CEBPD overexpression driven by amplified *CEBPD* is the consequence of amplified *MYC*-induced genome instability (38).

### The regulatory mechanism of CEBPD in UC progression

A high glucose demand and preferential reprogramming toward aerobic glycolysis are the major hallmarks of cancer progression (42). MYC is documented to potentiate aerobic glycolysis through the reinforcement of transcription activity on all glycolytic-related genes (43). The study by Chan et al. revealed that a novel positive feedback loop between CEBPD- and MYC-centric multilayered exists in UC to strengthen aerobic glycolysis: amplification of *MYC*-initiated chromosomal *CEBPD* instability to increase the CEBPD level. The overexpression of CEBPD protein further stabilized the MYC protein from proteasome-mediated degradation through transcription inhibition of *FBXW7* (F-box and WD repeat domain containing 7), a tumor suppressor serving as an E3 ubiquitin ligase of SCF (SKP1-CUL1-F-box protein) (44) to promote the level of solute carrier family 2 member 1 (SLC2A1; a central rate-limiting factor to regulate glucose transport in many cancers) and hexokinase II (HK2; a key mediator of aerobic glycolysis responsible for the aggressive phenotype) (45, 46). Subsequently, an increase in glucose uptake, lactate production, extracellular acidification rate (ECAR), and mitochondrial fragmentation/fission and a decrease in oxygen consumption rate (OCR), mitochondrial fusion type that were observed in CEBPD-overexpressing UC cell lines firmly proved the CEBPD-enforced metabolic conversion from mitochondrial oxidative phosphorylation to aerobic glycolysis.

Aside from employing MYC-dependent transcriptional regulation, CEBPD also utilizes an alternative pathway to coactivate aerobic glycolysis through transcriptional inhibition of hsa-miR-429 to boost HK2 expression (38). hsa-miR-429 belongs to the hsa-miR-200 family as a tumor suppressor in various cancers (47). The study by Chan et al. also disclosed the oncogenic characteristic of CEBPD on angiogenesis promotion through directly repressing the transcription of hsa-miR-429 to elevate the transcript of vascular endothelial growth factor A (VEGFA; a pivotal factor of angiogenesis and systemic metastasis) in UC cells (48, 49). Moreover, the upregulated expression of an angiogenesis-related gene called *MMP2* (matrix metalloproteinase-2) driven by *CEBPD* amplification has been identified and is remarkably associated with UC cell invasiveness (27).

Hyperactivated MAPK and PI3K/AKT/mTOR axis are pivotal signalings for cancer cell proliferation and survival (Makker et al., 2012). Hence, CEBPD likely raised cell viability and proliferation through the upregulated phosphorylation levels of MAPK3/1, PI3K, AKT1, mTOR, RPS6, and EIF4EBP1 in UC cell lines (38). The mTOR is one of the important glucose-sensing centers to adapt to the change in environmental glucose level. mTOR is activated and further hyperphosphorylates its downstream proteins to promote cell proliferation and metabolism regulation under glucose-sufficient conditions. Oppositely, glucose scarcity hampers mTOR activity and its anabolic processes to keep energy for cell survival (Leprivier and Rotblat, 2020). Of note, the influence of CEBPD on extreme glucose addiction to satisfy the substantial energy demand for abnormal anabolism responsive to hyperactivated mTOR pathway contrarily devastates glucose-deprived UC cells (38).

Clinical significance indicated that a high level of CEBPD has a positive correlation with the expression of MYC, HK2, VEGFA, and MMP2 and a negative association with hsa-miR-429 in UC patients. These genetic patterns also reflect the inferior survival outcome. Furthermore, abundant CEBPD synergized the mismanagement of glucose metabolism to augment the deteriorated effect on cancer aggressiveness and survival rate in UC patients with diabetes mellitus (DM; a disease with imbalanced glucose homeostasis accompanied by excessive glucose level in peripheral tissue) and SCID/beige mouse model with high-fat diet-induced DM. Summarily, CEBPD prompts the cancerization of the urothelium through the multi-manipulations on glycolytic metabolism shift and angiogenesis with CEBPD/MYC/HK2 axis, CEBPD/hsa-miR-429/HK2 axis, CEBPD/hsa-miR-429/VEGFA axis, and CEBPD/MMP2 axis (27, 38, 49).

### Characteristics of CEBPD responsive to cisplatin treatment in UC

At present, CDDP-based neoadjuvant chemotherapy (NAC) accompanied by radical cystectomy is regarded as a frontline treatment for patients with aggressive UC (50).

CDDP is a type of platinum-based antineoplastic medication. CDDP-DNA adduct formation for DNA damage and upregulation of reactive oxygen species (ROS) to lead to apoptosis are the foremost anticancer effects of CDDP (51, 52). Nevertheless, patients with cancer including UC usually have an excellent response to CDDP initially while suffering from relapse later on account of the CDDP resistance to decline clinical effectiveness (53, 54). The level of CEBPD has been reported to be upregulated after CDDP treatment. However, the side effect of CDDP-induced CEBPD on UC treatment is still ambiguous to be defined as dual functions to result in CDDP-induced chemotherapy resistance or drive CDDP-induced antitumorigenesis. To date, studies by Hour et al. (55) and Wang et al. (56) implicated that CEBPD possesses an oncogenic characteristic to promote CDDP-based chemotherapy resistance. The previous one showed that CDDP-induced CEBPD transcriptionally upregulates the level of Cu/Zn-superoxide dismutase (SOD1) to lower the level of ROS and apoptosis caused by CDDP treatment in UC. The later research indicated that the ATP-binding cassette (ABC) transporters (ABCB1, ABCC2) that function in multidrug resistance (MDR) in malignancy (57) are transcriptionally increased by EGFR/STAT3-driven CEBPD after CDDP treatment and has a high correlation with CDDP-related resistance in UC (55, 56). In contrast, the study by Lin et al. (58) pointed out that CEBPD-triggered hsa-miR-193b-3p directly targets cyclin D1 (CCND1) and ETS proto-oncogene 1 (ETS1) to cause cell cycle G1 arrest, invasive inhibition after CDDP treatment, suggesting that CDDP-induced CEBPD strengthens its tumor suppression.

Aside from the importance of CEBPD expression in cancer cells to tumor progression, the impact of non-cancer cells with CEBPD on the regulation of malignant aggressiveness has been paid close attention to recently. The tumor is a complex composed of cancer cells and tumor stroma including non-cellular [e.g., extracellular matrix (ECM)] and cellular components [e.g., activated cancer-associated fibroblasts (CAFs), mesenchymal stromal cells (MSCs), pericytes]. Tumor stroma is an essential part to support tumor growth and metastasis and even confers therapeutic resistance (59, 60). The study elucidated that substantial CEBPD is observed in the stromal compartments of PDAC and presents high relevance to the pancreatic cancer extravasation as well as metastasis but not tumor growth (61). In addition, treating breast cancer with CDDP promoted the level of CEBPD in CAFs as well as tumor-associated macrophages (TAMs) and led to chemoresistance through pentraxin 3 (PTX3)-induced invasion, metastasis, and stemness (62). Moreover, CDDP-induced CEBPD enforced the differentiation of fibroblasts toward myofibroblasts in the lung cancer microenvironment and activated the stromal cell-derived factor 4 (SDF4)/C-X-C motif chemokine receptor 4 (CXCR4) to trigger angiogenesis and distal metastasis (63). However, the contribution of systematic CEBPD-expressing tumor stroma and immune microenvironment to UC progression and its clinical relevance remain largely unknown; hence, more explorations are still necessary.

## Author contributions

Writing—original draft: T-CC. Writing—review and editing: C-FL, Y-LS. Funding acquisition: C-FL. Study supervision: C-FL, Y-LS. All authors contributed to the article and approved the submitted version.
